# The Prostate Care Questionnaire for Patients (PCQ-P): Reliability, validity and acceptability

**DOI:** 10.1186/1472-6963-9-199

**Published:** 2009-11-04

**Authors:** Carolyn Tarrant, Richard Baker, Andrew M Colman, Paul Sinfield, Shona Agarwal, John K Mellon, William Steward, Roger Kockelbergh

**Affiliations:** 1 Department of Health Sciences, University of Leicester, Leicester, UK; 2 School of Psychology, University of Leicester, Leicester, UK; 3 Department of Cancer Studies & Molecular Medicine, University of Leicester, Leicester, UK

## Abstract

**Background:**

In England, prostate cancer patients report worse experience of care than patients with other cancers. However, no standard measure of patient experience of prostate cancer care is currently available. This paper describes an evaluation of the reliability, validity and acceptability of the PCQ-P, a newly developed instrument designed to measure patient experience of prostate cancer care.

**Methods:**

The reliability, acceptability and validity of the PCQ-P were tested through a postal survey and interviews with patients. The PCQ-P was posted to 1087 prostate cancer patients varying in age, occupation, and overall health status, sampled from five hospitals in England. Nonresponders received one reminder. To assess criterion validity, 935 patients were also sent sections of the National Centre for Social Research Shortened Questionnaire; and to assess test-retest reliability, 296 patients who responded to the questionnaire were resent it a second time three weeks later. A subsample of 20 prostate cancer patients from one hospital took part in qualitative interviews to assess validity and acceptability of the PCQ-P. Acceptability to service providers was evaluated based on four hospitals' experiences of running a survey using the PCQ-P.

**Results:**

Questionnaires were returned by 865 patients (69.2%). Missing data was low across the sections, with the proportion of patients completing less than 50% of each section ranging from 4.5% to 6.9%. Across the sections of the questionnaire, internal consistency was moderate to high (Cronbach's alpha ranging from 0.63 to 0.80), and test-retest stability was acceptable (intraclass correlation coefficients ranging from 0.57 to 0.73). Findings on criterion validity were significant. Patient interviews indicated that the PCQ-P had high face validity and acceptability. Feedback from hospitals indicated that they found the questionnaire useful, and highlighted important considerations for its future use as part of quality improvement initiatives.

**Conclusion:**

The PCQ-P has been found to be acceptable to patients and service providers, and is ready for use for the measurement of patient experience in routine practice, service improvement programmes, and research.

## Background

In this paper we report the reliability and validity of a questionnaire to measure the experience of prostate cancer care. We also report the acceptability of the questionnaire to patients and to hospitals providing care for patients with prostate cancer.

In England, patients with prostate cancer report worse experience of care than patients with other cancers [1,2], and the provision of readily usable measures was one element of a wider initiative designed to improve services [3,4]. While care outcomes can be assessed through standard measures of health-related quality of life [5], which include reports of general health and impact on functioning, no standard measures of patient experience of the process and delivery of prostate cancer care are available [6]. In recent years, patient surveys have tended to focus on patient experience of care rather than on satisfaction with care. Satisfaction is a complex concept incorporating patients' expectations, their experience of what happened in care, their feelings about the care they received, and the importance to the patient of the aspect of care concerned. Measures of patients' experience - which comprise patients' reports of what happened in specific aspects of the delivery of care - are simpler to interpret and act upon than measures of satisfaction [7]. Surveys of experience are now generally preferred by providers, and considered helpful in evaluating and improving services.

In the wake of the NHS next stage review, all NHS organisations will be required to gather and publish information about patient experience of care, as part of a process of working towards high quality of care for all [8]. The availability of valid and acceptable measures of patient experience is crucial to ensuring that meaningful data are collected. To be suitable for wide use, measures of experience need to be developed systematically to address the issues that are important to patients, to be readily understood and acceptable by patients, and to meet standards of reliability and validity. They should also be acceptable to providers, enabling them to assess specific stages of care, depending on the provider's focus for quality improvement. Our aim was to develop a robust and acceptable measure suitable for use in routine practice and research. This paper describes such a measure of patient experience of prostate cancer care - the Prostate Care Questionnaire for Patients (PCQ-P) - and reports on the formal evaluation of the measure's reliability, validity and acceptability to patients and service providers. A companion measure for use with carers of men with prostate cancer has also been developed [9].

## Methods

### Development and characteristics of the PCQ-P

The PCQ-P is a measure developed from preliminary research designed to determine the issues most important to prostate cancer patients, including a literature review of the experiences of patients of prostate cancer care, and interviews with patients and service providers [10-12]. Key issues included: information and explanations, involvement in decision making, provision of advice and support, delays in care, choice, coordination of care, and practical issues (such as travel, and facilities at the hospital). Questions on these issues were developed, phrased in the words used by patients. Thorough piloting was undertaken [10]. The measure, along with a user guide, is available online for download and use [13]. The questionnaire is divided into five sections: GP visits and referral (Section A, 17 questions); tests at the hospital (Section B, 19 questions); diagnosis and treatment decision (Section C, 30 questions); treatment and discharge (Section D, 25 questions); and monitoring (Section E, 15 questions). There is a sixth section to collect health and sociodemographic information (Section F, 10 questions). Sections can be administered separately, or in appropriate combinations (e.g. it may be useful to give sections A, B, and C in combination to patients who have recently received a diagnosis of prostate cancer). A short version of the questionnaire, comprising 24 questions chosen to include the most important issues to patients, is also available [10,13].

### Sampling

Five hospitals in England were selected to participate in the study to test reliability, validity and acceptability to patients. Hospitals represented a range in terms of urban and rural locality, teaching and non-teaching hospitals, and foundation trust status (Table 1). Each hospital drew a consecutive sample of all patients who had been diagnosed with, or treated for, prostate cancer within the past two years, excluding patients who had died or were too ill to participate; this produced a list of between 152 and 253 patients per hospital depending on the numbers of patients with prostate cancer under their care. The sample included patients at different stages of care (e.g. undergoing treatment, undergoing post-treatment monitoring). A total of 1087 patients were identified. Hospital staff mailed sections of the PCQ-P to patients, and non-responders were sent one reminder. Sections A, B, C, and F in combination were sent to 431 patients (in hospitals 1 and 3), and Sections D, E and F to 504 patients (in hospitals 2 and 4). One hundred and fifty two patients (in hospital 5) received all 5 sections in combination.

**Table 1:** Features of hospitals: testing the questionnaire for reliability, validity, and acceptability to patients

	**Hospital 1**	**Hospital 2**	**Hospital 3**	**Hospital 4**	**Hospital 5**
**Foundation Trust**	No	No	No	Yes	Yes

**Teaching Hospital**	No	No	Yes	Yes	Yes

**Population served**	Urban	Rural	Urban	Urban	Rural

**Location in England**	South	South West	South West	London	East Anglia

### Measures of reliability, validity and acceptability to patients

To assess criterion validity, 935 patients (in hospitals 1 to 4) were also sent sections of the National Centre for Social Research Shortened Questionnaire (NCSRSQ), a questionnaire designed to measure patients' experience of care for several different forms of cancer [14]. To assess test-retest reliability, 296 patients from two hospital sites (hospitals 1 and 2) were posted the PCQ-P questionnaire again three weeks later.

In addition, 20 patients from hospital 5, who had completed all sections of the PCQ-P, took part in semi-structured interviews to explore acceptability and face validity. Eleven face-toface and nine telephone interviews were carried out. Interviews were not transcribed, but notes were taken by the interviewer during the interview.

Key properties of the questionnaire sections, including aspects of validity, reliability, and acceptability to patients were analysed. For the purpose of testing the properties of the questionnaire, overall scores were calculated for each section of the questionnaire, by summing scores across questions and converting to a score out of 100, with higher scores indicating more positive experiences of care [10]. All statistical analyses were conducted using SPSS version 16.0.

#### Acceptability to patients

Acceptability was evaluated by examining completion rates for individual questions and questionnaire sections, and by analysing distributions of responses for individual questions. Acceptability was also assessed in patient interviews, by asking patients how they felt about the experience of completing the questionnaire.

#### Validity

Criterion validity was assessed by examining Pearson correlations between scores on sections B to E of the PCQ-P and the NCSRSQ. The NCSRSQ did not contain comparable questions for section A of the PCQ-P. The NCSRSQ is a measure of problems in care, where in contrast to the PCQ-P a higher score indicates a less positive experience of care, therefore a negative correlation between the scores on the two questionnaires was expected. The NCSRQ is a generic questionnaire rather than being specific to prostate cancer, and a medium-sized correlation co-efficient (-0.3 to -0.5) was expected as an indicator of validity [15,16]. Face validity was investigated through patient interviews, in which patients were asked to describe their experiences of care in conjunction with inspection of their responses to the questionnaire, as well as being asked directly whether there were any important aspects of care that were not included in the questionnaire. Content validity was assessed through comparing the results of exploratory principal components analysis (PCA) with themes identified through the preliminary research [11,12].

#### Reliability

Internal consistency reliability for each section was measured using Cronbach's alpha [17]. Stability reliability was assessed using intraclass correlation coefficients (ICCs) between scores on the first and second completion of the questionnaire. Stability was also assessed by calculating the percentages of patients answering each question the same way on the first and second completion of the questionnaire.

### Usability and acceptability to service providers

The usability and acceptability of the questionnaire to service providers was assessed through inviting a separate sample of hospitals to coordinate and run a patient experience survey using the PCQ-P, then seeking feedback on their experiences of this process. Hospitals were recruited via Service Improvement Leads (SILs) at the Cancer Networks, who identified hospitals within their network that would be willing to take part in this stage of the study. Four hospitals in England were selected from the list of identified hospitals, selected to ensure a range in terms of urban and rural locality, teaching and non-teaching hospitals, and foundation trust status. The characteristics of the four hospitals which took part in this separate stage of acceptability testing are given in Table 2. Hospitals were provided with questionnaires, a user guide developed as part of the study [13], and software to enable them to enter their data and produce basic summary results. Three hospitals each used a different selection of sections of the full measure, and one hospital used the short version of the questionnaire covering the whole patient journey. Hospitals were asked to survey around 100 patients, and feedback on their experiences was gained through semi-structured interviews with one or two key persons who had administered the survey in each hospital (total of 5 interviews), along with informal discussion with other members of hospital staff. Interviews were not transcribed, but notes were taken by the interviewer during the interview.

**Table 2:** Features of hospitals: testing the questionnaire for usability and acceptability to service providers

	**Hospital A**	**Hospital B**	**Hospital C**	**Hospital D**
**Foundation Trust**	Yes	Yes	No	No

**Teaching Hospital**	Yes	No	No	Yes

**Population served**	Urban	Rural	Rural	Urban

**Location in England**	London	South West	Midlands	Midlands

## Results

### Acceptability to patients

Questionnaires were returned by 865 patients (69.2%); 355 completed Sections A, B, C and F (response rate: 61%), and 510 completed Sections D, E and F (response rate: 77.7%). This response rate is similar to that achieved for prostate cancer patients in the National Survey of NHS Cancer Patients in 1999/2000 [18]. The demographic characteristics and health status of responders are summarised in Table 3.

**Table 3:** Demographic characteristics and overall health status of patient sample: reliability and validity testing

**Age (years)**	***N *(%)^i^**
< 54	18 (2.1)

55-64	215 (24.9)

65-74	350 (40.5)

75+	262 (30.3)

**Overall health**	

Very good	253 (29.2)

Good	385 (44.5)

Fair	166 (19.2)

Poor	28 (3.2)

Very poor	10 (1.2)

**Ethnicity**	

White British/Irish	803 (92.8)

South Asian	10 (1.2)

African/Caribbean	17 (2.0)

Other	2 (0.2)

**Current situation**	

Employed	185 (21.3)

Retired	624 (72.1)

Other	24 (2.8)

**Current or most recent occupation**	

Professional	239 (27.6)

Managerial	178 (20.6)

Clerical	35 (4.0)

Technical/craft	148 (17.1)

Manual/service	136 (15.7)

^i^May not add to 100 due to missing data

The proportion of patients completing fewer than 50% of the questions in each section was low: 16 patients (4.5%) for Section A, 20 (5.6%) for Section B, 18 (5.1%) for Section C, 30 (5.9%) for Section D, and 35 (6.9%) for Section E. Missing data were usually due to patients omitting whole sections, sometimes appropriately (e.g. no medical tests at the named hospital) but without making the reasons clear. For patients who completed more than 50% of the questionnaire, missing data for individual questions ranged from 0% to 15.4%, the majority showing less than 10% missing data. Responses to most questions were well distributed across response options. Overall, patients more often reported positive experiences, with mean overall scores ranging from 65.9 to 86.4 across the sections. Descriptive statistics of section scores are shown in Table 4.

**Table 4:** Descriptive statistics of overall scores from the five sections of the questionnaire

**Section^i^**	***N***	**Mean** **score**	***SD***	**Minimum**	**Maximum**	**% with lowest possible score**	**% with highest possible score**
**Section A**	307	65.9	21.1	.00	100.0	0.7	5.1

**Section B**	328	82.3	14.0	28.5	100.0	0.0	3.9

**Section C**	292	86.4	13.4	27.8	100.0	0.0	17.5

**Section D**	304	71.0	16.2	16.2	100.0	0.0	1.2

**Section E**	459	71.3	20.8	.00	100.0	0.4	12.0

^i^Section A = GP visits and referral, Section B = tests at the hospital, Section C = diagnosis and treatment decision, Section D = treatment and discharge, Section E = monitoring

In the patient interviews, patients described the questions as easy to understand, and the majority did not report any problems with filling in the questionnaire. One patient commented that his case did not fit well with the sequence of sections as they were presented in the full questionnaire (compiled from all five subsections), and two patients felt that the full questionnaire was too long. Three patients emphasised that each section should be administered as soon as possible after the relevant phase of treatment or care, as they found it difficult to remember in detail events from several months previously. Patients suggested that the sections should be divided and used as separate questionnaires, or with just two to three sections in combination, with appropriate sections being administered as soon as possible after the relevant stage of care.

### Criterion validity

Out of 935 patients, 592 (63.3%) completed both a PCQ-P and a NCSR questionnaire. Response rates were 224/431 (52%) for Sections A, B, C, and F, and 368/504 (73%) for Sections D, E, and F. Pearson's correlation coefficient between Sections B and C and the first half NCSRSQ was -0.23 (*p *= 0.002, *N *= 175), and between Sections D and E and the second half NCSRSQ was -0.46 (*p *< 0.001, *N *= 201). These correlations are in the expected direction, the first is small and the second is medium [15,16], and both are significant at *p *< 0.005.

### Face and content validity

All interview participants indicated that the questionnaires covered important aspects of care. However, several patients highlighted gaps in the section on discharge. The particular issues identified as important were: knowing what to expect in terms of recovery time and side effects; and knowing how to obtain appropriate supplies after discharge (such as continence pads). This led to two questions on these issues being added to the questionnaire.

Content validity was assessed through exploratory principal components analysis. Analysis identified three to four components for each section of the questionnaire (see additional file 1: exploratory PCA). For example in section A (GP visits and referral), three components emerged, and inspection of questions within each component suggested that the components related to: 'explanation', 'taking the problem seriously' and 'experience of referral'. Comparison of these components with themes from the initial patient interviews and literature review [11,12] confirmed that the key aspects of care identified in the preliminary research were satisfactorily incorporated in the PCQ-P.

### Internal consistency reliability

Cronbach's alpha coefficients ranged from 0.63 to 0.80, indicating moderate to high internal consistency for all sections of the PCQ-P (Table 5).

**Table 5:** Reliability: Internal consistency and stability of the five sections of the PCQ-P

	**Internal consistency**	**Stability: Test-retest reliability**		
**Section^i^**	Cronbach's α	1st mailing mean score *SD*, min-max (*N*)	2nd mailing mean score *SD*, min-max (*N*)	Intraclass Correlation Coefficient (ICC)

**Section A**	0.80	69.1	68.5	0.68
		19.6, 11.5-100 (60)	18.7, 16.7-100 (62)	

**Section B**	0.63	84.4	81.3	0.57
		15.3, 28.5-100 (73)	10.6, 39.7-99.4 (72)	

**Section C**	0.77	88.4	87.6	0.61
		12.0, 55.0-100 (62)	13.1,31.3-100 (63)	

**Section D**	0.80	73.2	74.8	0.73
		17.1, 27.7-99.23	14.5, 41.4-100 (49)	
		(48)		

**Section E**	0.68	74.3	74.9	0.70
		21.9, 16.7-100 (61)	19.7, 18.8-100 (60)	

^i^Section A = GP visits and referral, Section B = tests at the hospital, Section C = diagnosis and treatment decision, Section D = treatment and discharge, Section E = monitoring

### Test-retest reliability

Out of 296 patients, 148 (50%) completed the retest questionnaire; 79/125 (63.2%) completed Sections A, B, C, and F, and 69/171 (40.4%) completed Sections D, E, and F. Patients completing retest questionnaires did not differ significantly from other patients in terms of age, health status, ethnic group, or employment status (*p *> 0.05 in each case). The test-retest ICCs for the five sections were between 0.57 and 0.73, and all were significant at *p *< 0.001 (Table 5), indicating acceptable reliability [19]. The consistency of responses to individual questions was high, between 52.6% and 100% of patients answering identically on the first and second mailing. Most questions (94, 88.7%), were answered perfectly consistently by over 70% of responders. The questions where responses were less consistent were those with a higher number of response options, and for these questions the difference between responses on the first and second completion of the questionnaire tended to be a shift to the neighbouring response option, for example, from 'good' to 'very good'.

### Acceptability to service providers

Three of the four participating hospitals chose to administer the questionnaire by post, and one used a combination of postal administration and face-to-face administration in urology clinics. None of the hospitals reported major difficulties with running the survey. However, the administration of the survey was seen as time consuming, and hospitals found this problematic; one hospital in particular had difficulty finding staff with time available to run the survey. Hospitals felt that the provision of support to run the survey would be helpful, and one hospital suggested that administering the survey prospectively to patients over a longer period of time would be more manageable and would produce valuable data.

Agreement to conduct the survey had initially been obtained from a consultant in each hospital, and some staff felt that an essential first stage would be a meeting involving all staff who would have a role in the administration of the survey. This would promote ownership of the survey, ensure local support, and provide a setting where input of time could be negotiated. There were mixed feelings about the involvement of an external organisation to coordinate the survey, possibly on a national basis; staff described a preference for using surveys as part of their own internal quality improvement process, and were resistant to the idea of surveys being used as an "external stick" (Hospital B) to raise performance, or being linked to financial incentives.

**Figure 1:**
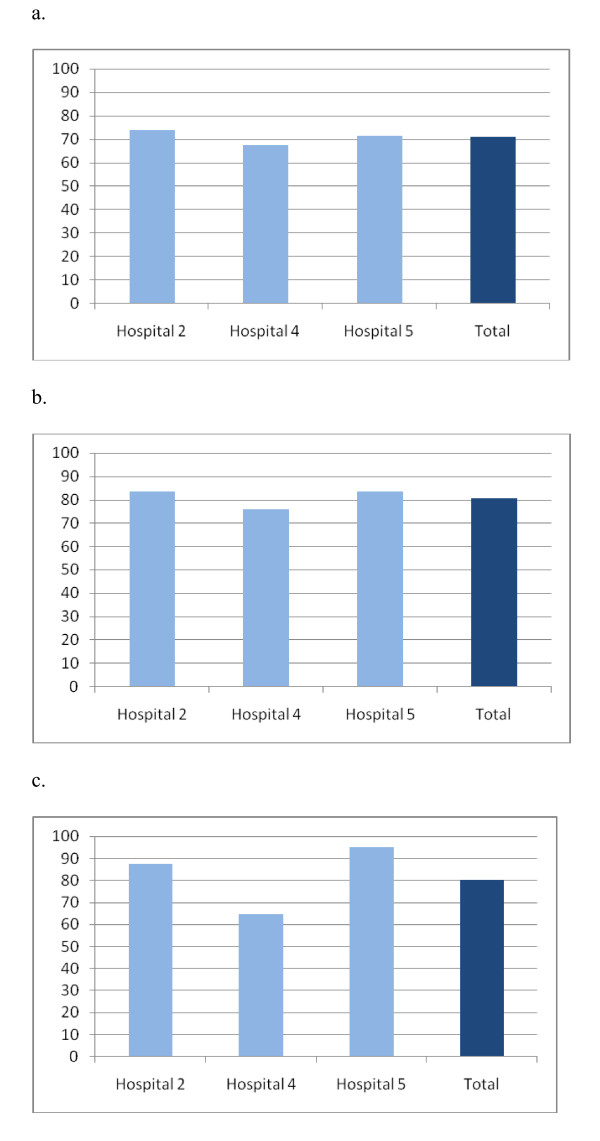
**Comparative data from three hospitals**. a. Overall scores for section D. b. Scores for component 'discharge'. c. Percentage of patients responding positively to 'discharge' question: 'Did the doctor or nurse give you any information about who to contact for advice or support (e.g. specialist nurse, patient support group)?'

Hospital staff feedback indicated that they found the questionnaire relevant and would value it as a tool for their own use, particularly if benchmarks or comparative data were available from other hospitals. Staff were particularly keen to have access to comparative data for local hospitals, as they felt this would have the greatest impact on efforts to improve quality - they "like to do better than others locally!" (Hospital A). It is possible to present comparative data on the PCQ-P at the level of whole sections, components within sections, and individual questions, using bar charts (Figure 1 illustrates this with data from three of the hospitals involved in reliability and validity testing). Although further work is required to establish the components within each section, the PCQ-P shows potential to produce data across a range of levels of detail that may be useful in interpreting their results. For example, inspecting results at the level of section and component scores allows a hospital to identify areas where they may be performing less well than other hospitals, then inspecting specific question results within a component can help pinpoint specific areas of care which may be problematic.

## Discussion

The evaluation of the PCQ-P demonstrates that the instrument incorporates issues that are important to prostate cancer patients, has good reliability and validity, and is acceptable to patients and service providers. The PCQ-P can be used flexibly - single sections can be used independently or in combination with other relevant sections, and a short version [10] covering the whole patient journey is also available.

Some limitations should be noted. The evaluation of criterion validity was hampered by the absence of any satisfactory criterion. No other suitable measure of patient experience of prostate cancer care was available, hence a generic patient experience questionnaire (NCSRSQ) was used as an indicative criterion. Nevertheless, the findings of the criterion validity check show small to medium sized, but significant, correlations between scores on the PCQ-P and the NCSRSQ. Taken in conjunction with other tests of the PCQ-P's properties, this provides support for the validity of the PCQ-P as a measure of patient experience of prostate cancer care. The findings of this study confirm that the PCQ-P is suitable for use in service improvement programmes. The study also highlights the approach to using the questionnaire preferred by patients (sections of the questionnaire administered separately, as soon as possible after the corresponding care stage), and indicates important considerations for hospitals planning to use the questionnaire, including ensuring that staff time is made available to administer the questionnaire. The development of a version of the questionnaire that could be self-administered via computer would be valuable in addressing this problem.

Attempts to develop and improve services should include assessment of patient experience when evaluating the impact of changes. In the UK, the greater involvement of commissioners in the future will present an additional opportunity to monitor and improve patient experience, and patient experience surveys should play a role in this [20]. The PCQ-P could be used in future national patient surveys. The use of a robust and acceptable questionnaire, specifically designed to collect experiences of prostate cancer care, would allow GPs and hospitals to evaluate their performance on the aspects of care that are most important to prostate cancer patients, and to compare their performance to other, similar GP practices and hospitals. The establishment of benchmark scores for the questionnaire would be of value in this context, and could be established via a national survey.

## Conclusion

This study has demonstrated how a sophisticated patient experience questionnaire can be developed and evaluated systematically. Although the development process is prolonged, thoroughness in development and testing provides confidence in the data produced by the measure, and increases the value of the data for use in service evaluation and research. The PCQ-P could provide a starting point for the development of instruments for other cancer groups, as many of the issues that it covers are relevant to other cancers. For example, the need for information and support throughout care is pertinent to the other common cancers, including lung, colorectal, and breast cancer. New measures would require careful testing before use. Nonetheless, the detailed process undertaken to develop the prostate care questionnaire provides a springboard for developing instruments for other cancers.

## Competing interests

The authors declare that they have no competing interests.

## Authors' contributions

CT participated in the design of the study, carried out the research and data analysis, and prepared the first draft of this paper; RB was lead of the complete project, and participated in the design of the study reported in this paper; AMC participated in the design of the study and in data analysis. PS participated in the design of the study and carried out the research. SA carried out the research. JKM, WS, and RK participated in the design of the study and advised on study conduct. All authors were involved in revising and approving the final manuscript.

## Pre-publication history

The pre-publication history for this paper can be accessed here:



## Supplementary Material

Additional file 1 **Exploratory Principal Components Analysis with Varimax rotation for each section of the PCQ-P**. Table showing results of an exploratory PCA for all sections of the PCQ-P. Click here for file

## References

[B1] Airey C, Becher H, Erens B, Fuller E (2002). National Survey of NHS Patients - Cancer: National Overview 1999/2000.

[B2] National Audit Office (2004). National Patient Survey (Cancer).

[B3] Department of Health (2004). The NHS Cancer Plan and the new NHS: Providing a patientcentred service.

[B4] Department of Health (2007). Cancer Reform Strategy.

[B5] Ware JE, Sherbourne CD (1992). The MOS 36-item short-form health survey (SF-36): I. Conceptual framework and item selection. Med Care.

[B6] McNaughton M, Collins M, Walker-Corkery E, Barry MJ (2004). Health-related quality of life, satisfaction and economic outcome measures in studies of prostate cancer screening and treatment 1990-2000. J Natl Cancer I Monogr.

[B7] Coulter A (2005). Opinion and Experience: Do They Concur?.

[B8] Darzi A (2008). High Quality Care for All.

[B9] Sinfield P, Baker R, Tarrant C, Agarwal S, Colman AM, Steward W, Kockelbergh R, Mellon JK Development of measures of patient and carer experience of prostate cancer care. 3. Reliability, validity and acceptability of the Prostate Care Questionnaire for Carers (PCQ-C). BMC Health Serv Res.

[B10] Baker R, Sinfield P, Agarwal S, Tarrant C, Mellon K, Steward W, Colman AM, Kockelbergh R, Sproston K (2007). Prostate Cancer Care: Improving Measures of the Patient Experience Report for the National Co-ordinating Centre for NHS Service Delivery and Organisation R&D. London: NCCSDO.

[B11] Sinfield P, Baker R, Agarwal S, Tarrant C (2008). Patient-centred care: what are the experiences of prostate cancer patients and their partners?. Patient Educ Couns.

[B12] Sinfield P, Baker R, Camosso-Stefinovic J, Colman AM, Tarrant C, Mellon JK, Steward W, Kockelbergh R, Agarwal S (2009). Men's and carers' experiences of care for prostate cancer: a narrative literature review. Health Expect.

[B13] Prostate cancer care questionnaires and user guide. http://www.sdo.nihr.ac.uk/files/adhoc/77-questionaires2.pdf.

[B14] Prescott A (2004). National Survey of NHS Patients - Cancer: Analysis of Themes.

[B15] Cohen J (1992). A power primer. Psych Bull.

[B16] Cohen J, Hillsdale NJ (1988). Statistical power analysis for the behavioral sciences.

[B17] Cronbach LJ (1951). Coefficient alpha and the internal structure of tests. Psychometrika.

[B18] Department of Health Methodology of the national survey of NHS cancer patients. http://www.dh.gov.uk/en/Publicationsandstatistics/PublishedSurvey/NationalsurveyofNHSpatients/Nationalsurveycancer/DH_4001299.

[B19] Pasta DJ, Suhr D (2004). Creating scales from questionnaires: PROC VARCLUS vs. factor analysis. Proceedings of the Twenty-Ninth Annual SAS Users Group International Conference (Paper 205-29).

[B20] Department of Health (2007). World Class Commissioning: Competencies.

