# Supplementary material for: The Prostate Care Questionnaire for Patients (PCQ-P): Reliability, validity and acceptability
Source: BMC Health Serv Res. 2009 Nov 4;9:199. doi: 10.1186/1472-6963-9-199 (PMC2777154; doi:10.1186/1472-6963-9-199)
Supplement: Additional file 1 — Exploratory Principal Components Analysis with Varimax rotation for each section of the PCQ-P. Table showing results of an exploratory PCA for all sections of the PCQ-P. [file 1472-6963-9-199-S1.docx]

Additional file 1: Exploratory Principal Components Analysis with Varimax rotation for each section of the PCQ–P^i^

**SECTION A: GP VISITS & REFERRAL**

|  | **Component 1: Explanation** | **Component 2: Experience of referral** | **Component 3:**  **Taking the problem seriously** |
| --- | --- | --- | --- |
| *A5. Did the doctor or nurse explain that the tests were trying to find out whether you might have prostate cancer?* | .791 |  |  |
| *A10. Did the doctor or nurse clearly explain your test results?* | .737 |  | .344 |
| *A7. Did the doctor or nurse explain what would happen if the results were abnormal?* | .730 |  |  |
| *A14. Did the doctor or nurse explain that you were being referred to hospital to find out if you had prostate cancer?* | .697 |  |  |
| *A6. Did the doctor or nurse explain how the DRE test would be carried out / that the PSA test is not always reliable?* | .645 |  |  |
| *A9. Were you given a choice about whether you wanted to be tested for prostate cancer?* | .597 | .323 |  |
| *A11. Did the doctor or nurse give you your test results in a considerate way?* | .571 |  | .507 |
| *A15. Did the doctor or nurse give you a choice of which hospital you wanted to go to / the date you wanted to be seen on?* |  | .718 |  |
| *A16. Were you asked if you had any needs when the referral arrangements were made (e.g. transport needs, time of appointment)?* |  | .718 |  |
| *A17. Did the doctor or nurse tell you how soon you would be seen at the hospital?* |  | .605 |  |
| *A8. Did the doctor or nurse offer you any written information about the test(s)?* |  | .588 |  |
| *A3. Did the doctor or nurse take your concerns seriously?* |  |  | .704 |
| *A13. How did you feel about the time the GP’s practice/local assessment centre took to refer you to hospital?* |  |  | .701 |

**SECTION B: TESTS AT THE HOSPITAL**

|  | **Component 1: Explanation & support** | **Component 2: Quality of care** | **Component 3: Appointment** |
| --- | --- | --- | --- |
| *B14. Did the doctor or nurse clearly explain to you how long you would have to wait for your test results?* | .664 |  |  |
| *B13. Did the doctor or nurse explain that you may need medication (e.g. antibiotics) to control any infections caused by the biopsy?* | .648 |  |  |
| *B16. Did the doctor or nurse clearly explain to you what would happen next?* | .628 |  |  |
| *B9. Did the doctor or nurse explain that these test results were to find out if you had prostate cancer?* | .531 |  |  |
| *B15. Did the doctor or nurse offer you any support while you were waiting for your test results (e.g. someone to talk to about any concerns that you had)?* | .505 |  |  |
| *B12. When you had your biopsy were you offered a local anaesthetic?* | .434 |  | .340 |
| *B7. How would you rate: Waiting area; Availability of refreshments; Toilets; Rooms where the tests were carried out* |  | .833 |  |
| *B10a. Explanation of what test would involve: TRUS/biopsy; urine flow; PSA; DRE; Scans (MRI, CT)* |  | .722 |  |
| *B6. Did you experience any problems with your hospital visit(s)?* |  | .483 |  |
| *B11. Did the doctor or nurse explain to you that the biopsy might be painful?* |  | .386 |  |
| *B8. Did you have enough privacy while the doctor or nurse was examining/testing you?* |  |  | .783 |
| *B5. How did you feel about the length of time you had to wait for your first appointment for tests at the hospital?* |  |  | .440 |
| *B3. Were you advised that it might be helpful if someone (e.g. wife/partner, relative) could attend the hospital appointment when you went for your tests?* | .333 | .396 | -.404 |

**SECTION C: DIAGNOSIS AND TREATMENT DECISION**

|  | **Component 1: Explanation & support** | **Component 2: Making treatment decision** | **Component 3: Getting the diagnosis** | **Component 4: Length of wait** |
| --- | --- | --- | --- | --- |
| *C16. Did the doctor or nurse clearly explain what these treatment options would involve?* | .817 |  |  |  |
| *C17. Did the doctor or nurse explain the possible side effects or consequences of these treatment options?* | .795 |  |  |  |
| *C18. Did the doctor or nurse clearly explain what could be done about the side effects?* | .641 | .318 |  |  |
| *C28. Did the doctor or nurse give you the information about who to contact for advice or support (e.g. specialist nurse, patient support group, charity)?* | .636 |  |  |  |
| *C19. Did the doctor or nurse clearly explain why the other treatment options were not suitable for you?* | .608 | .358 |  |  |
| *C24. Did the doctor or nurse involve you as much as you wanted in the decision about which treatment to have?* | .591 |  |  |  |
| *C6. Did the doctor or nurse clearly explain your diagnosis?* | .511 |  |  |  |
| *C25. Were you confident that the treatment decision was the best one for you?* | .475 |  |  |  |
| *C27. Did the doctor or nurse tell you that you could change your mind about which treatment to have?* |  | .826 |  |  |
| *C26.* *After the treatment decision had been made did the doctor or nurse tell you that you could discuss your treatment decision again?* |  | .803 |  |  |
| *C22. Did the doctor or nurse encourage you to take your time before making a decision about which treatment to have?* | .415 | .520 |  |  |
| *C13. Were you given any written information about your diagnosis?* |  | .517 |  |  |
| *C4. Were you advised that it might be helpful if someone (e.g. partner, relative) could attend the hospital appointment with you to get your diagnosis?* |  | .473 |  |  |
| *C9. Did the doctor or nurse clearly explain how aggressive the cancer was likely to be?* | .349 | .367 |  |  |
| *C20. Did the doctor or nurse give you any written information about the treatment options / the possible side effects or consequences of the treatment options?* |  | .360 |  |  |
| *C7. Were you given your diagnosis in a considerate way?* |  |  | .835 |  |
| *C5. Did you have enough privacy when you discussed your diagnosis?* |  |  | .809 |  |
| *C3. How did you feel about the length of the time you had to wait to get your diagnosis?* |  |  | .483 |  |
| *C15.How did you feel about the length of time between being given your diagnosis and discussing your treatment options?* |  |  |  | .754 |
| *C21. How do you feel about the length of time between being given your treatment options and discussing your treatment decision?* |  |  |  | .697 |
| *C12. How did you feel about the length of time you had to wait to discuss your diagnosis with the specialist nurse?* |  |  |  | .694 |

**SECTION D: TREATMENT AND DISCHARGE**

|  | **Component 1: Preparation for discharge** | **Component 2: Treatment** | **Component 3: Information** |
| --- | --- | --- | --- |
| *D15. Before you left hospital or finished treatment did the doctor or nurse explain to you what would happen next (e.g. arrangements for follow-up)?* | .730 |  |  |
| *D18. Were you given equipment or supplies (e.g. continence pads) to use at home to help you care for yourself?* | .698 |  |  |
| *D17. Did the doctor or nurse discuss with you how to manage any potential side effects of the treatment (e.g. continence, problems with sex, pain)?* | .674 |  |  |
| *D16. Did the doctor or nurse give you any information about who to contact for advice or support (e.g. specialist nurse, patient support group)?* | .647 |  |  |
| *D14. Did the doctor or nurse explain how well the treatment was going/had gone?* | .606 |  |  |
| *D20. Did a doctor or nurse organise the aftercare services that you needed (e.g. district nurse, physiotherapist)?* | .605 |  | .333 |
| *D9. Did you experience any problems with your hospital visit(s)?* | .359 |  |  |
| *D10.How would you rate: the treatment; the nursing; the food/drink; the ward (e.g. privacy, noise, cleanliness)?* |  | .730 |  |
| *D12. While you were being treated, do you think that the hospital staff did everything they could to help with your pain or discomfort (e.g. give you enough medication)?* |  | .638 |  |
| *D6. Before you started your treatment, did a doctor or nurse give you information about the treatment to help you feel prepared (e.g. what your treatment would involve, what you should/should not do during your treatment)?* | .335 | .526 |  |
| *D23. Have staff in different places worked well together when caring for you for this condition: between GP’s practice and hospital; between hospital and hospital; between different departments (e.g. Urology and Oncology)?* |  | .505 |  |
| *D5. How did you feel about the length of time you had to wait for your treatment to start?* |  | .462 |  |
| *D8. While you were receiving treatment were you able to discuss any concerns about your treatment with the doctor or nurse?* | .314 | .324 |  |
| *D21. Did the doctor or nurse offer you any financial information on welfare or benefits?* |  |  | .673 |
| *D19. Did a doctor or nurse discuss whether you might need any extra day to day help (e.g. help with housework)?* |  |  | .660 |
| *D13. Did the doctor or nurse give you any information about complementary therapies (e.g. diet/diet supplements/acupuncture/massage/reflexology)?* |  | .345 | .571 |
| *D7.Were you advised that it might be helpful if someone (e.g. partner, relative) could go with you when you went for treatment?* |  |  | .487 |

**SECTION E: MONITORING**

|  | **Component 1: Explanation & reassurance** | **Component 2:**  **Advice** | **Component 3:**  **Choice** |
| --- | --- | --- | --- |
| *E9. Does the doctor clearly explain what the test results mean?* | .769 |  |  |
| *E3. Did the doctor or nurse explain why you have these regular tests?* | .706 |  |  |
| *E8. Has the doctor or nurse clearly explained what can affect your PSA levels (e.g. exercise, ejaculation)?* | .552 | .338 |  |
| *E5. Has a doctor or nurse reassured you that the length of the wait between these tests for prostate cancer is appropriate for you?* | .528 |  |  |
| *E10. Has the doctor or nurse given you a telephone number to ring if you need any help or advice, or have any questions about your condition (e.g. for talking to a specialist nurse)?* |  | .860 |  |
| *E11. Do you know how to get advice and help in managing symptoms or side effects of treatment (e.g. continence, problems with sex, pain)?* |  | .785 |  |
| *E12. Are staff in different places working well together when monitoring you for this condition: between GP’s practice and hospital; between hospital and hospital; between different departments (e.g. Urology and Oncology)?* | .403 | .416 |  |
| *E6. Have you been offered a choice of where to have these tests (e.g. GP’s practice, hospital)?* |  |  | .800 |
| *E7. Were you offered a choice of how to be given your test results (e.g. face-to-face, by telephone)?* |  |  | .761 |

^i^Number of components limited based on examination of eigenvalues and scree plots. Loadings of 0.3 and higher only shown
